# Evaluation of a ConvitVax/anti-PD-1 combined immunotherapy for breast cancer treatment

**DOI:** 10.18632/oncotarget.27283

**Published:** 2019-11-12

**Authors:** María José Godoy-Calderón, Eglys González-Marcano, Jeismar Carballo, Ana Federica Convit

**Affiliations:** ^1^ Unidad Experimental de Inmunoterapia, Fundación Jacinto Convit, Caracas, Venezuela; ^2^ Jacinto Convit World Organization, Inc., Palo Alto, CA, USA

**Keywords:** combination immunotherapies, cancer immunotherapy, breast cancer, autologous tumor cells vaccine, anti-PD-1

## Abstract

Breast cancer therapies using checkpoints alone have not been highly effective. Based on previous experiences using the ConvitVax, an autologous tumor cells/bacillus Calmette-Guérin (BCG)/formalin-based vaccine, in breast cancer and the potential success of combined therapies, we sought to ascertain whether the ConvitVax combined with anti-PD-1 enhances the antitumor effect in a 4T1 breast cancer model. Animals received four weekly injections of either PBS (G1), ConvitVax (200 μg cell homogenate, 0.0625 mg BCG, 0.02% formalin) (G2), 50 μg anti-PD-1 (G3), or ConvitVax plus anti-PD-1 (200 μg cell homogenate, 0.0625 mg BCG, 0.02% formalin, 50 μg anti-PD-1) (G4). Five weeks post tumor induction all mice were euthanized, tumors extracted and evaluated pathologically and by immunohistochemistry. The combination group (G4) showed 10% more tumor necrosis, greater infiltration of PD-1^+^ cells and lower infiltration of TAMs, evidencing that the combination of ConvitVax and anti-PD-1 can improve the antitumor effect of the vaccine. Using a higher anti-PD-1 dose and administering each treatment at different times could further potentiate the effect of our therapy. Given the vaccine’s low cost and simple preparation, its use in combination with checkpoints or other target-specific compounds may lead to a highly effective personalized breast cancer immunotherapy.

## INTRODUCTION

Immunotherapy has emerged in the last decade as the most promising approach to cancer treatment with lower side effects than conventional chemotherapy and radiotherapy. The most commonly used immunotherapies are vaccines and checkpoint inhibitors. Checkpoint molecules are critical components of T-cell activation and immune regulation. One example are cell surface receptors, known as programmed cell death protein 1 (PD-1), which when upregulated in T cell accompanying cancer cells may allow them to escape antitumor immunity. The ligand of PD-1 receptors, the programmed death-ligand 1 (PD-L1), is expressed in a variety of epithelial cancers. These changes in the PD-1/PD-L1 signaling pathway may be contributing to the maintenance of an immunosuppressive tumor microenvironment [1].

The success of anti-PD-1 immunotherapies in the treatment of melanoma [2] and non-small cell lung cancer [3] have led to its approval by the FDA. However, it has not been as effective in other tumor types. For example, recent clinical trials of patients with metastatic triple-negative breast cancer found equivalent median progression-free survival (PFS) with anti-PD-1 monotherapy relative to historical chemotherapy controls, with only 19–21% patients showing overall response [4–6]. On the other hand, the combination of immune checkpoint blockade with conventional cancer treatments, molecularly targeted therapies or other immunotherapies have shown to be a promising strategy to potentiate its efficacy in breast cancer, though requiring further research to effectively identify who will respond to these immunotherapies [7, 8]. This indicates that for breast cancer the therapeutic benefit is limited to a number of patients and that combination therapies need to be investigated [9]. In concordance with this trend on combined immunotherapies, two large randomised trials are currently assessing the efficacy of drugs targeting PD-1 (NCT03036488 and NCT02954874), in combination with standard neo-adjuvant (preoperative) or adjuvant (postoperative) chemotherapies in early-stage triple-negative breast cancer [8].

Cancer vaccines are known to induce a specific immune response against tumor cells and establish long-term immune memory response, thus preventing tumor recurrence while reducing the likelihood of toxic side effects [10]. The little efficacy of anti-PD-1 monotherapy observed in patients with metastatic breast cancer is partly due to the low number of tumor-infiltrating lymphocytes in most breast cancers [8]. Recently, we showed the effectiveness and ability to induce a significant antitumor cell infiltration by a polyvalent vaccine composed of autologous tumor cells, bacillus Calmette-Guérin (BCG) and formalin in a breast cancer murine model, henceforth referred to as “ConvitVax” [11]. Pre-clinical and clinical studies combining tumor vaccines with checkpoint inhibitors have shown a significant enhancement of the vaccine’s induced immune response and antitumor effects [12–14]. In order to ascertain whether checkpoint inhibition could add to our prior polyvalent vaccine results, we evaluated in a murine model the antitumor effect of a combination of ConvitVax with monoclonal anti-PD-1 antibody. We tested whether the vaccine response, mainly represented by a marked infiltration of cytotoxic cells, can be enhanced by inhibiting a possible immune suppression mediated by the PD-1 pathway.

## RESULTS

### Combination of ConvitVax and anti-PD-1 treatment (G4) enhances tumor elimination without improvement in tumor arrest

**Figure 1:**
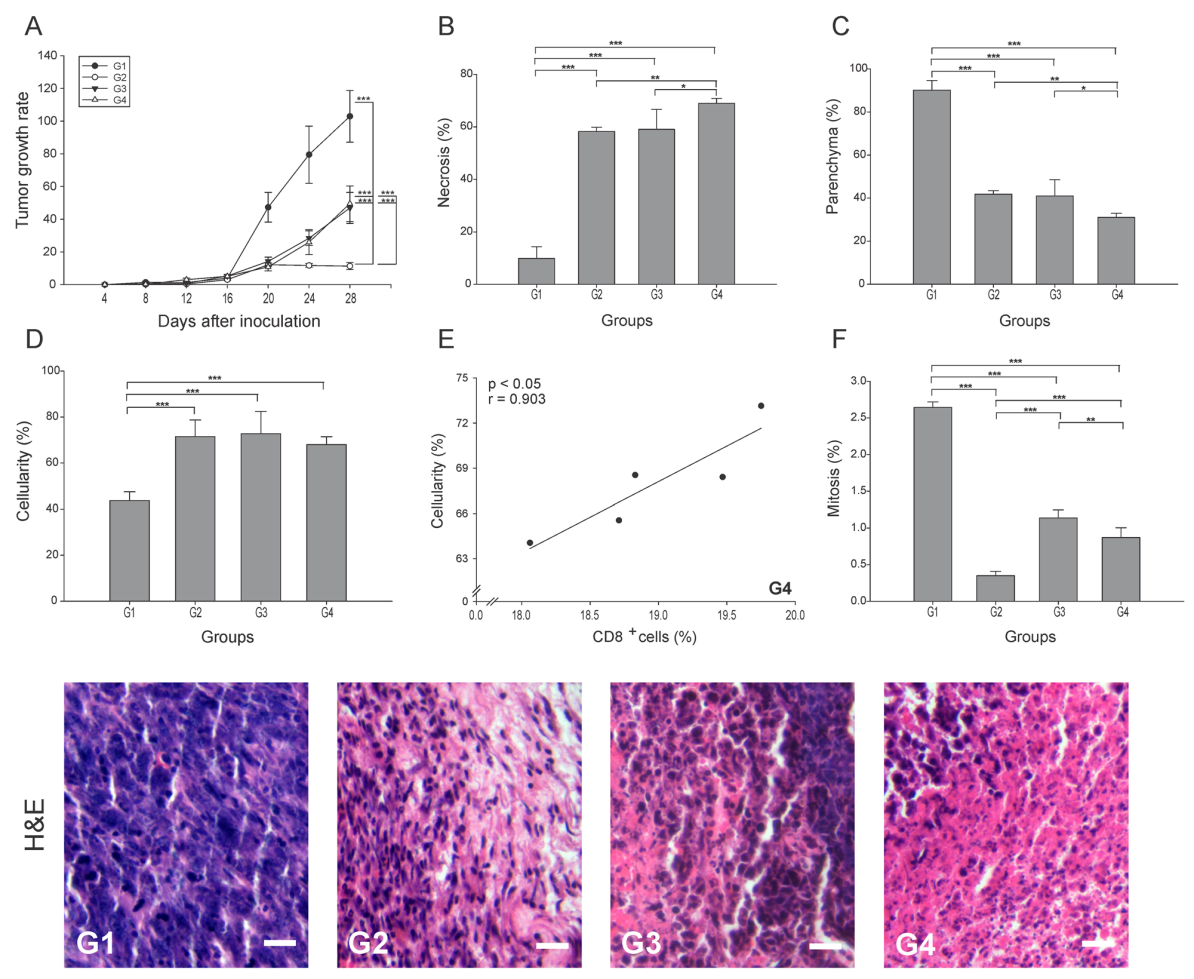
ConvitVax/anti-PD-1 combined treatment enhances tumor elimination without tumor arrest improvement in 4T1 tumors Baseline tumor volume was measured 4 days post inoculation (tumor induction) with tumor volume measured every 4 days subsequently until 28 days post tumor induction. **(A)** Tumor growth rate for each group presented as a percentage of volume increase relative to the initial volume by days after tumor induction. **(B)** Tumor necrosis percentage was determined, resulting G4 with the highest value. **(C)** The parenchyma percentage diminished in all treated groups being significantly lower in G4. **(D)** The cellularity percentage was markedly increased in all treated groups compared to G1. **(E)** Scatter diagram analysis showing a positive correlation between CD8^+^ T cells and percentage of cellularity in G4. **(F)** Significant decrease of mitosis percentage in G2 compared to all groups. Tumor slides obtained from each mice group were stained with H&E to determine the percentage of necrosis, parenchyma, cellularity and mitosis. Representative H&E-stained slides for each group are presented. Scale bar, 10 μm. All data is shown as the mean percentage ± SEM of five mice per group. Tukey’s post-hoc test results are shown (^*^p ≤ 0.05; ^**^p ≤ 0.01; ^***^p ≤ 0.001).

To determine the effect of each treatment on tumor progression, the tumor growth rate was calculated for all groups. Our results indicate that the addition of anti-PD-1 showed a 2-fold reduction (p ≤ 0.05) for G3 and G4, whereas G2 showed an 11-fold reduction compared to G1 (Figure 1A). However, when evaluating necrosis, we observed an elimination of nearly 70% of the tumor tissue in G4, which was higher than G3 and G2, and 59% higher than G1 (p ≤ 0.05) (Figure 1B). Also, as expected from the level of necrosis, G4 showed a 3-fold decrease in the percentage of parenchyma compared to G1 (p ≤ 0.05), while G2 and G3 showed only a 2-fold decrease (Figure 1C). A marked infiltration of cells with morphological characteristics of immune cells was also seen in all treated groups, with a cellularity of approximately 50% higher than G1 (p ≤ 0.05) (Figure 1D). The increased cellularity observed in G4 might be composed of antitumor cells as suggested by its strong positive correlation with CD8^+^ T cells (r = 0.903, p ≤ 0.05) (Figure 1E). Additionally, we determined a reduction in the percentage of mitosis, which may be indicative of tumor arrest. Relative to G1, the most significant decrease of the mitotic index was observed in G2, being 7.5 times lower than G1 (Figure 1F). G3 and G4 showed mitotic indexes 2.3 and 3 times lower than G1, respectively (p ≤ 0.05) (Figure 1F). In general, these results might suggest that the combination of ConvitVax and anti-PD-1 (G4) does not significantly potentiate the tumor elimination promoted by the vaccine, and has a lower ability to control tumor cells division under the treatment schedule implemented, in comparison with the vaccine treatment itself. However, the combined immunotherapy induced the highest level of necrosis, which might be affecting the reduction of the tumor size. Therefore, the apparent non-regression of the tumors might not be due to a progression of the disease but to the presence of a highly necrotic tissue and a high infiltration of immune cells.

### Antitumor immune cell infiltration

**Figure 2:**
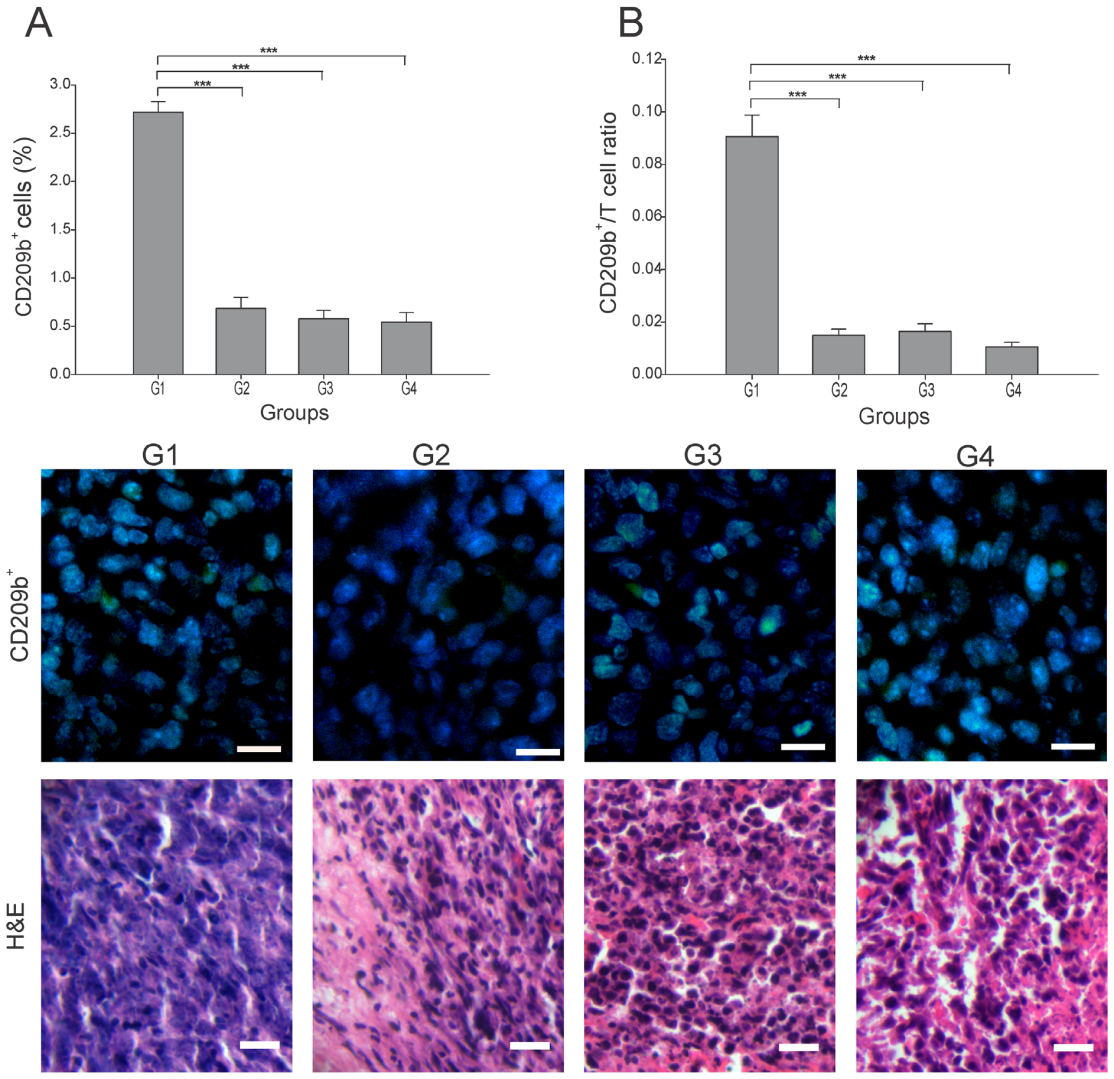
Effect of each treatment over APCs infiltration Tumor slides obtained from each mice group were processed by immunohistochemistry to detect CD209b^+^ cells. **(A)** CD209b^+^ cell count significantly decreased in G2-G4, compared to G1. Representative immunohistochemical staining of CD209b^+^ cells in all four groups studied are presented as CD209b cells in green and DAPI nuclear staining in blue. Representative H&E stained slides for each group are presented. Scale bar, 10 μm. **(B)** Dynamic changes of CD209b^+^ to CD8^+^ T cell ratios were analyzed based on the proportions of CD209b^+^ and CD8^+^ T cells. The data is shown as the mean ± SEM of five mice per group. Tukey’s post-hoc test results are shown (^***^p ≤ 0.001).

Antigen-presenting cells (APCs), including dendritic cells (DCs), play an essential role in the first step of the antitumor immune response through the processing of the dying cancer cells. Subsets of tumor DCs, depending on their maturation/functional status, have varying capacity for cross-presentation and immunogenicity [15]. T-cell interactions with immature DCs can lead to T-cell tolerance through various mechanisms [16]. We found that in G2-G4 the number of APCs (CD209b^+^ cells) was 4 to 5 times lower than in G1 (p ≤ 0.05) (Figure 2A). To estimate the T cells priming by APCs, the APC/T cell ratio was calculated using the quantitation of CD209b^+^ cells as APCs. A low APC/T cell ratio would suggest efficient T cells activation and expansion, while high APC/T cell ratio may indicate an arrest of T cell expansion [17]. We observed a low APC/T cell ratio in all three treated groups, which contrasted to the 9-fold higher ratio seen in G1 (p ≤ 0.05) (Figure 2B). Although a specific determination of DCs maturation status could not be performed in this study, some calculated correlations provide an estimate of the prevalent DCs subsets in the tumor microenvironment. Thus, a prevalence of mature DCs is suggested in G3 by the high negative correlation of CD209b^+^ cells with CD8^+^ T cells (r = - 0.895, p ≤ 0.05) (Supplementary Figure 1); perhaps the low percentage of CD209b^+^ cells was at the expense of mature DCs that led to lymphocyte priming. Hence, no additive enhancement of T cell priming was observed when applying the combination treatment under this treatment schedule.

**Figure 3:**
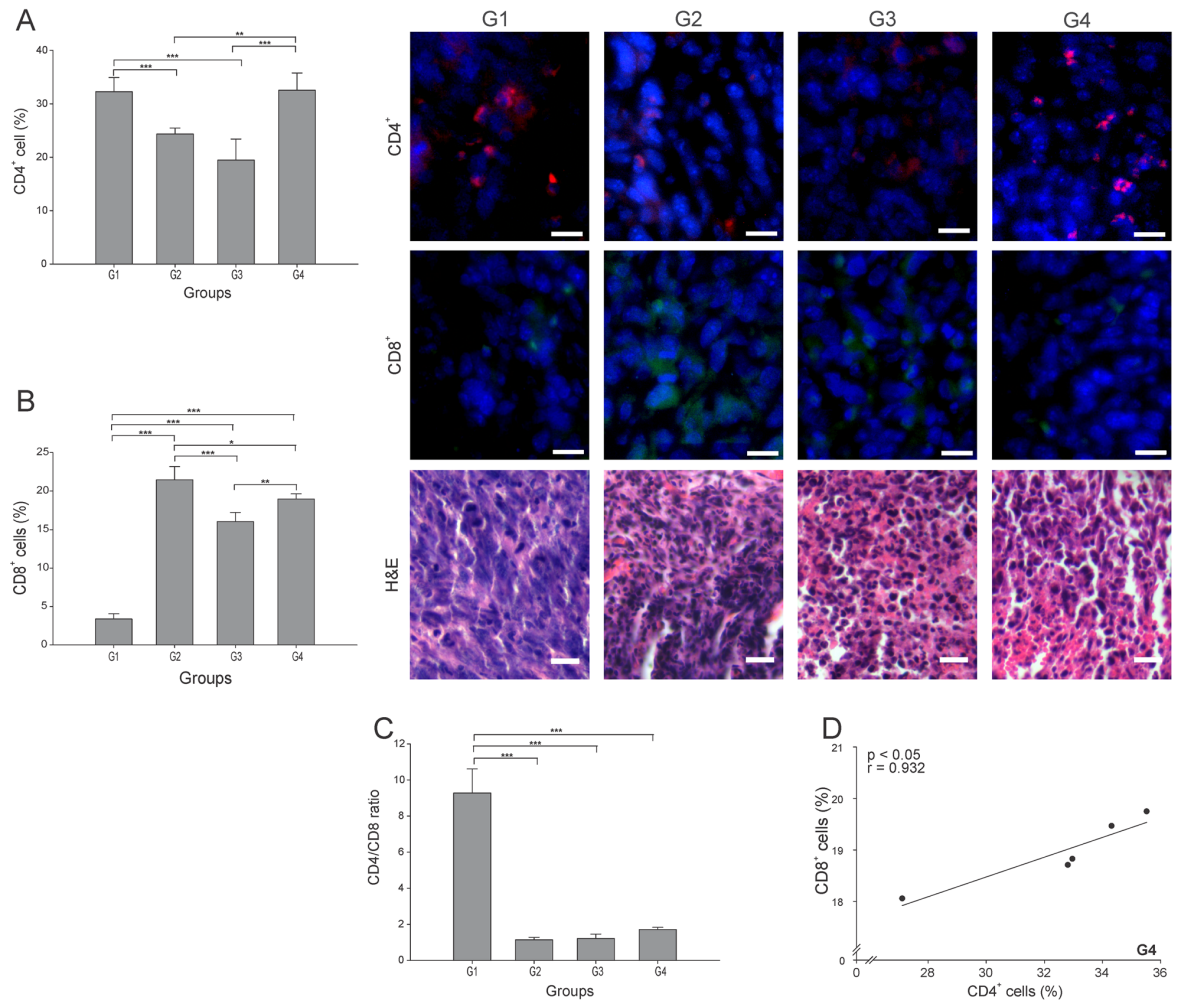
ConvitVax/anti-PD-1 combined treatment promotes CD8^+^ T cells infiltration and possible prevalence of CD4^+^ Th1 cells Tumor slides obtained from each mice group were processed by immunohistochemistry to determine CD4^+^ and CD8^+^ T cells. **(A)** CD4^+^ T cell counts significantly diminished in G2 and G3 compared to G1. G4 CD4^+^ T cell count was not statistically different from G1. Representative immunohistochemical staining of CD4^+^ T cells in all four groups studied are presented as CD4^+^ T cells in red and DAPI nuclear staining in blue. Scale bar, 10 μm. **(B)** CD8^+^ T cell counts significantly increased in G2-G4 compared to G1. Representative immunohistochemical staining of CD8^+^ T cells in all four groups studied are presented as CD8^+^ T cells in green and DAPI nuclear staining in blue. Scale bar, 10 μm. Representative H&E stained slides for each group are presented. Scale bar, 10 μm. **(C)** Dynamic changes of CD4^+^ to CD8^+^ T cell ratios were analyzed based on the proportions of CD4^+^ and CD8^+^ T cells. **(D)** Scatter diagram analysis showing a positive correlation between CD8^+^ and CD4^+^ T cells in G4. The data is shown as the mean percentage ± SEM of five mice per group. Tukey’s post-hoc test results are shown (^*^p ≤ 0.05; ^**^p ≤ 0.01; ^***^p ≤ 0.001).

Among the most important cells in tumor elimination are CD4^+^ T cells, particularly the Th1 subtype, whereas the CD4^+^ Tregs (regulatory T cells) subtype have been implicated in tumor progression and maintenance of a pro-tumor microenvironment by interfering in CD8^+^ T responses [18]. We quantitated the total of CD4^+^ T cells infiltrating each tumor, and found that G4 showed the highest percentage, albeit not statistically different from G1 (p ≥ 0.05) (Figure 3A). G2 and G3 showed nearly 75% and 60% less CD4^+^ T cells than G1, respectively (p ≤ 0.05) (Figure 3A). CD8^+^ T cells play a central role in cancer immunity through their capacity to kill malignant cells upon recognition by T-cell receptor (TCR) of specific antigenic peptides [20]. Although G2 presented the highest percentage of CD8^+^ T cells, G4 showed a significant high value, almost 6-times higher than G1 (Figure 3B). We could not conduct staining of maturation markers in CD4^+^ T cells. Thus, to indirectly estimate the subtype of CD4^+^ T cells present, we calculated the CD4/CD8 ratio. High CD4/CD8 ratios have been associated to worse prognosis in patients with different types of cancer, including breast cancer [18, 19], suggesting higher proportion of CD4^+^ Treg cells. Here we found a CD4/CD8 ratio between 5 to 8 times lower in G2-G4 compared to G1 (p ≤ 0.05) (Figure 3C). Additionally, considering the important role of CD4^+^ T cells in the CD8^+^ T cell’s activation, a strong positive correlation between these two lymphocytes subtypes (r = 0.932, p ≤ 0.05) (Figure 3D) was observed in G4. In order to identify the precise proportion of the CD4^+^ T cell sub-populations, additional experiments should be performed.

**Figure 4:**
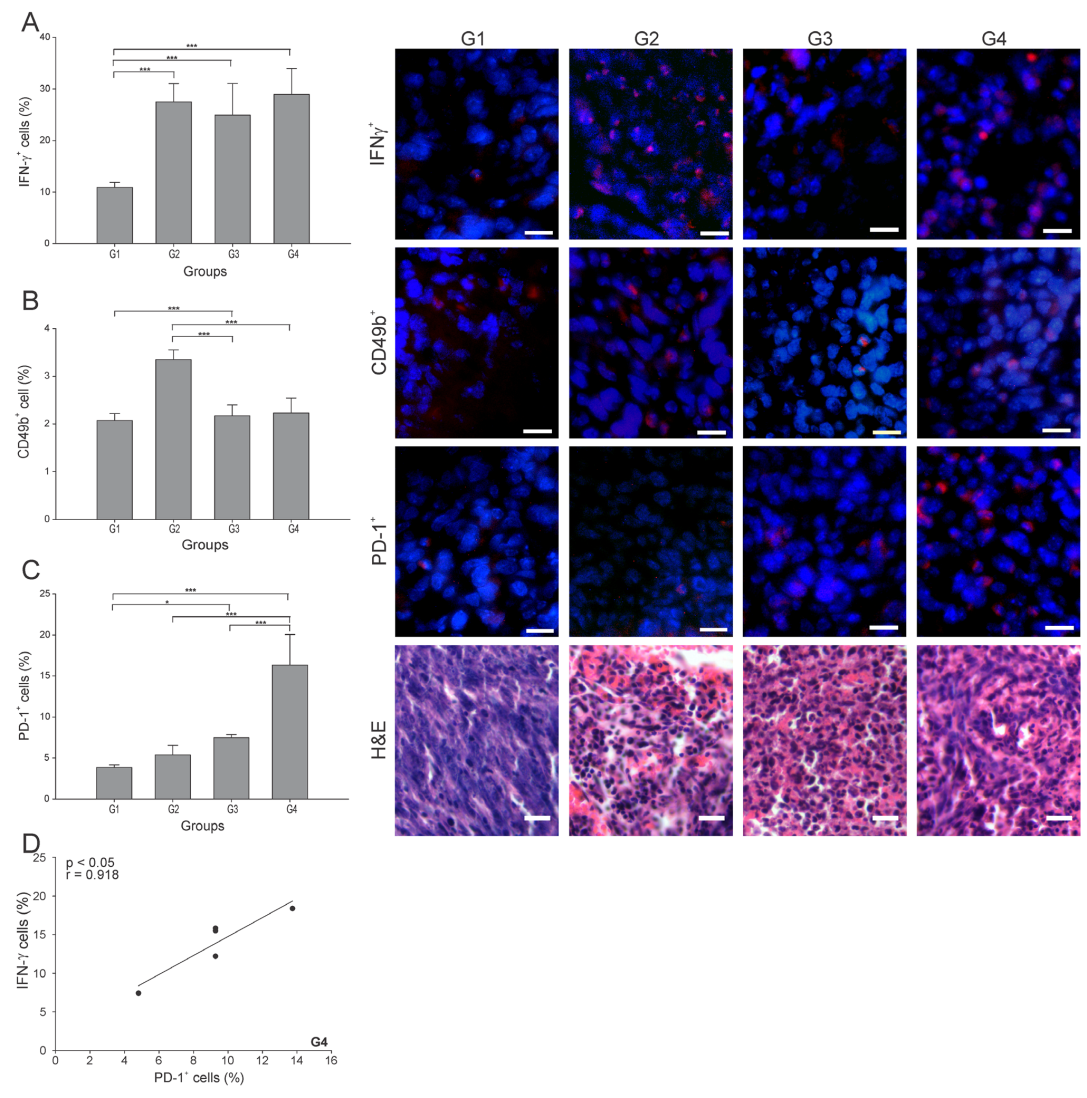
ConvitVax/anti-PD-1 combined treatment induces a marked PD-1^+^ cells infiltration in 4T1 tumors Tumor slides obtained from each mice group were processed by immunohistochemistry to determine IFN-γ^+^, NK (CD49b^+^) and PD-1^+^ cells. **(A)** IFN-γ^+^ cell counts significantly incremented in all treated groups compared to G1. Representative immunohistochemical staining of IFN-γ^+^ cells in all four groups studied are presented as IFN-γ^+^ cells in red and DAPI nuclear staining in blue. Scale bar, 10 μm. **(B)** NK (CD49b^+^) cell counts incremented in all treated groups compared to G1, having an almost twice increment of the NK cell count in G2. Representative immunohistochemical staining of CD49b^+^ cells in all four groups studied are presented as CD49b^+^ cells in red and DAPI nuclear staining in blue. Scale bar, 10 μm. **(C)** PD-1^+^ cell counts were significantly high in all treated groups compared to G1. G4 showed the highest percentage of PD-1^+^ cells. Representative immunohistochemical staining of PD-1^+^ cells in all four groups studied are presented as PD-1^+^ cells in red and DAPI nuclear staining in blue. Scale bar, 10 μm. Representative H&E stained slides for each group are presented. Scale bar, 10 μm. **(D)** Scatter diagram analysis showing a positive correlation between PD-1^+^ cells and IFN-γ^+^ cells in G4. The data is shown as the mean percentage ± SEM of five mice per group. Tukey’s post-hoc test results are shown (^*^p ≤ 0.05; ^**^p ≤ 0.01; ^***^p ≤ 0.001).

IFN-γ plays a critical role in regulating T cell activation, including driving Th1 immune responses required for tumor rejection [21]. The percentage of IFN-γ^+^ cells was more than 2.5 times higher in all three treated groups than in G1 (p ≤ 0.05) (Figure 4A), with no difference among them. The pattern was somewhat different for Natural Killer cells (NK cells) (CD49b^+^ cells), with the cell count roughly the same in G3 and G4 (Figure 4B), but with G2 showing almost twice the NK cell count compared to G1.

PD-1 is widely expressed on several immune cells and it remains upregulated during initial T-cell activation [22]. Here, G4 showed the highest percentage of PD-1^+^ cells, being 4, 3 and 2 times higher than G1, G2 and G3, respectively (Figure 4C). Interestingly, we observed a strong positive correlation between PD-1^+^ and IFN-γ^+^ cells in G4 (r = 0.918, p ≤ 0.05) (Figure 4D).

**Figure 5:**
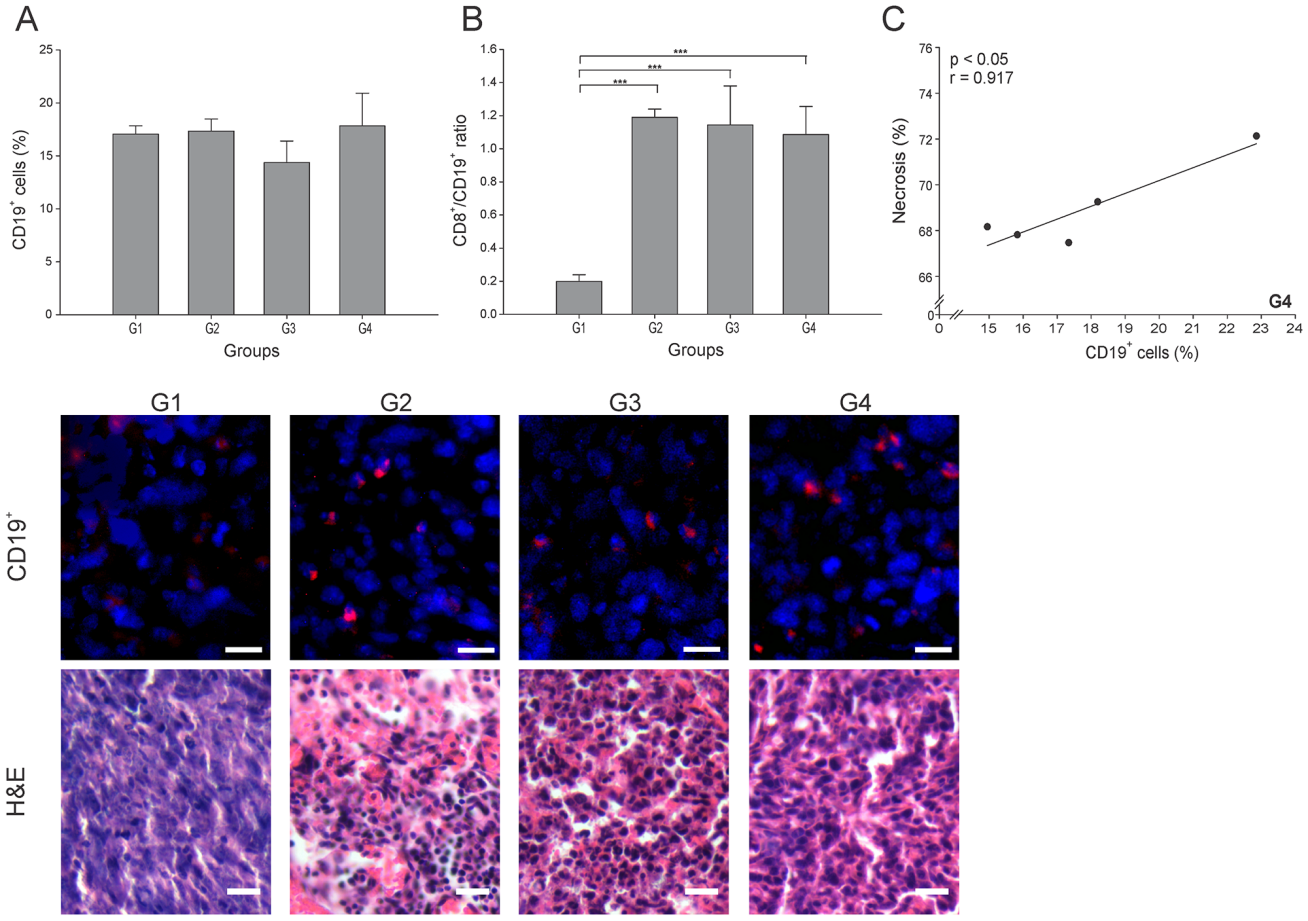
ConvitVax/anti-PD-1 combined treatment effect over B cells Tumor slides obtained from each mice group were processed by immunohistochemistry to determine B cells (CD19^+^). **(A)** CD19^+^ cell counts did not show significant changes between all groups. Representative immunohistochemical staining of CD19^+^ cells in all four groups studied are presented as CD19^+^ cells in red and DAPI nuclear staining in blue. Scale bar, 10 μm. Representative H&E stained slides for each group are presented. Scale bar, 10 μm. **(B)** Dynamic changes of CD19^+^ to CD8^+^ T cell ratios were analyzed based on the proportions of CD19^+^ and CD8^+^ cells. **(C)** Scatter diagram analysis showing a positive correlation between CD19^+^ cells and necrosis percentage in G4. The data is shown as the mean ± SEM of five mice per group. Tukey’s post-hoc test results are shown (^*^p ≤ 0.05; ^**^p ≤ 0.01; ^***^p ≤ 0.001).

Besides the significant antitumor role played by cytotoxic cells, B cells can also inhibit tumor development through interaction with tumor tissue, secretion of antibodies and cytokines, and antigen presentation [23]. However, regulatory B cells subtype (Bregs) have been involved in tumor progression [24]. Surprisingly, all groups showed similar CD19^+^ cell (B cells) percentages (p ≥ 0.05) (Figure 5A). Hence, it was essential to calculate the CD8^+^ T cells/B cells ratio (CTL/B ratio) to infer the nature of the B cells in each group. High CTL/B ratios would suggest CD8^+^ T cells stimulation by effector B cells leading to tumor elimination [25]. In accordance with an important cellular antitumor effect, all three (3) treated groups showed a CTL/B ratio 10 times higher than G1 (p ≤ 0.05) (Figure 5B). A significantly positive correlation between CD19^+^ cells and necrosis percentage (r = 0.917, p ≤ 0.05) was found in G4 (Figure 5C). Additional specific cell identification experiments should be performed to accurately determine the subset of B cells infiltrating the tumor microenvironment.

In summary, the combined treatment (G4) induced a higher infiltration of PD-1^+^ cells, though with a lower infiltration of NK cells, compared to the ConvitVax alone (G2).

### The combination of ConvitVax and anti-PD-1 (G4) significantly reduces tumor associated macrophages (TAMs) tumor infiltration

**Figure 6:**
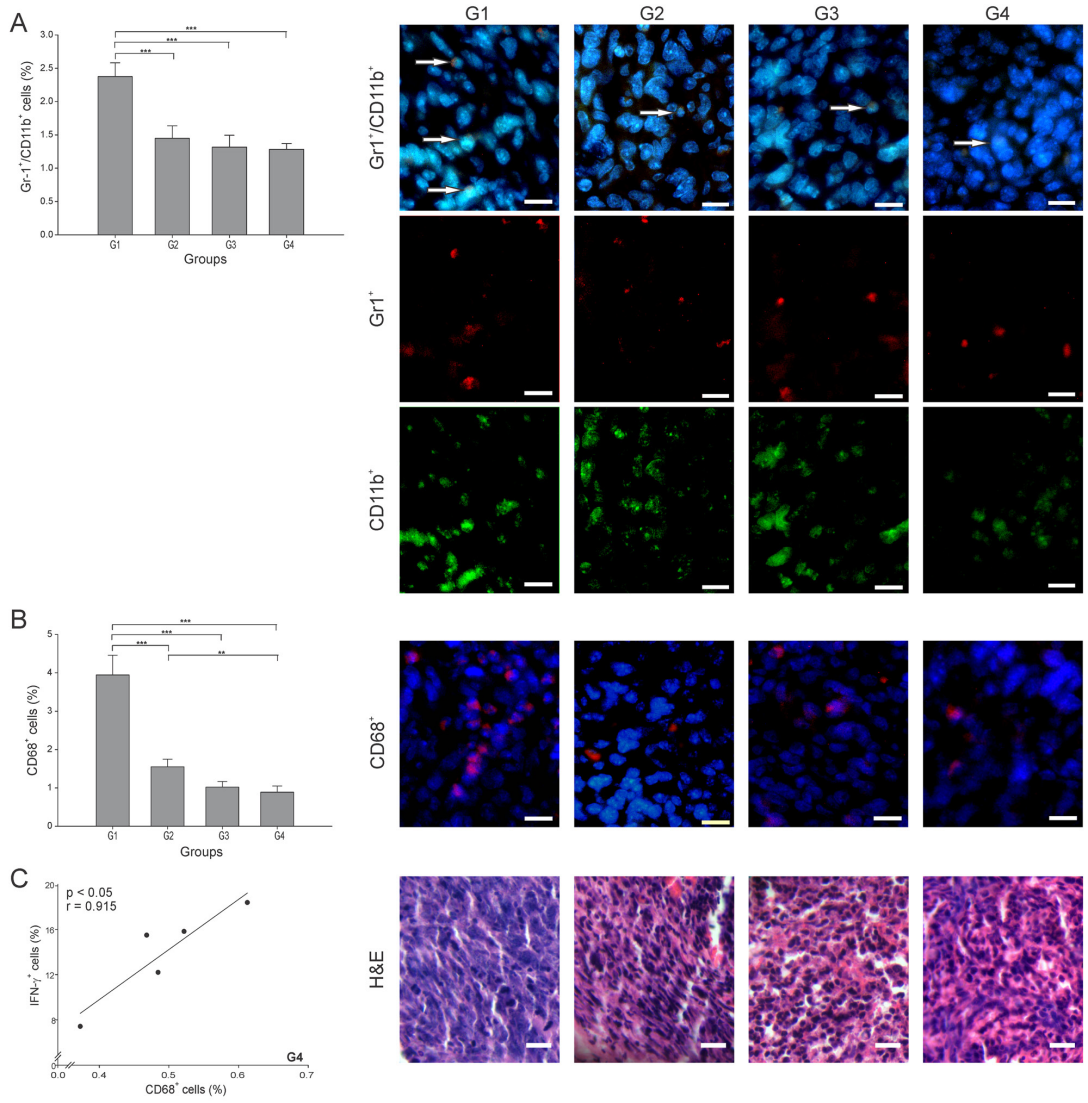
ConvitVax/anti-PD-1 combined treatment reduces TAMs and MDSCs tumor infiltration Tumor slides obtained from each mice group were processed by immunohistochemistry to determine Gr-1^+^/CD11b^+^ cells (MDSCs) and CD68^+^ (TAMs). **(A)** Gr-1^+^/CD11b^+^ cell counts were significantly low in G2-G4 compared to G1. Representative immunohistochemical staining of Gr-1^+^/CD11b^+^ cells in all four groups studied are presented as Gr-1^+^ cells in red, CD11b^+^ cells in green, Gr-1^+^/CD11b^+^ cells in yellow (arrows) and DAPI nuclear staining in blue. Scale bar, 10 μm. **(B)** CD68^+^ cell counts significantly diminished in all treated groups compared to G1, with G4 showing the lowest value. Representative immunohistochemical staining of CD68^+^ cells in all four groups studied are presented as CD68^+^ cells in red and DAPI nuclear staining in blue. Scale bar, 10 μm. Representative H&E stained slides for each group are presented. Scale bar, 10 μm. **(C)** Scatter diagram analysis showing a positive correlation between CD68^+^ and IFN- γ^+^ cells in G4. The data is shown as the mean percentage ± SEM of five mice per group. Tukey’s post-hoc test results are shown (^*^p ≤ 0.05; ^**^p ≤ 0.01; ^***^p ≤ 0.001).

Two of the most studied anti-inflammatory and pro-tumor cells are the Gr-1^+^/CD11b^+^ (MDSCs) and CD68^+^ (TAMs) cells. To consider an anti-cancer treatment successful, the levels of these two cell types should be meaningfully reduced. In this study, the number of MDSCs was lowered significantly in all treatment groups, nearly 50% lower than in G1 (p ≤ 0.05) (Figure 6A). There is a subtype of TAMs cells known as TAMs M1, which are characterized by a capacity to secrete IFN-γ and promote tumor elimination, whereas TAMs M2 promote tumor progression through the secretion of anti-inflammatory cytokines [26]. The TAMs percentage also decreased in all groups, interestingly with G4 showing the lowest value, almost 5 times lower than G1 (p ≤ 0.05) (Figure 6B). Additionally, a high positive correlation of TAMs and IFN-γ^+^ cells (r = 0.915, p ≤ 0.05) was found in G4 (Figure 6C). Overall, an important indicator of effectiveness in G4 was the diminution of TAMs, considering that these cells are one of the main immune suppressors that prevent the establishment of an effective immune antitumor response.

## DISCUSSION

In the last decade various immunotherapies have been successful in treating different types of cancer. To date, no immunotherapeutic approach has been found truly effective for breast cancer. Based on the proved success of checkpoint inhibitors, the previously demonstrated antitumor effects of ConvitVax [11] and the potential benefits of using combined therapies [2, 3, 27], we evaluated the effectiveness and possible mechanism of a combined therapy with our vaccine and anti-PD-1.

Overall, we may say that the combined therapy (G4) did not significantly improve the general antitumor effect seen with ConvitVax alone (G2). However, very important antitumor features were improved with the combined therapy, which are relevant to mention and give way to more specific studies to continue evaluating this combination. The first feature observed is the high infiltration of CD4^+^ T cells, though not directly addressing whether this infiltration is due to CD4^+^ Th1 cells or Tregs. Interestingly, G4 showed a very similar CD4/CD8 ratio as that reported for G2, with G4 showing a lower CD8^+^ T cell count. A specific identification of each CD4^+^ T cell lineage should be performed to determine if the high count of CD4^+^ T cells in G4 contain a small number of Tregs that slightly diminishes the recruitment capacity of CD8^+^ T cells into the tumor, lowering its total count. On the other hand, the correlation between CD4^+^ and CD8^+^ T cells in G4 might be suggestive of the presence of CD4 Th1 cells in this group. This is compatible with a Th1 immune response initiated due to a correct priming of T cells by APCs, which guides T cells to generate an efficient tumor elimination [28], as it was evidenced by the high necrosis percentage measured in G4.

The second feature of this comparison is the percentage of TAMs, which in G4 was almost 50% lower than that reported for G2. Considering that the anti-PD-1 may induce M1 polarization of TAMs [29], the extensive tumor elimination plus the positive correlation of TAMs and IFN-γ^+^ cells in G4, we can suggest that the diminution of TAMs might be at the expense of TAMs M2 reduction. It is known that IFN-γ overcomes TAM-induced immunosuppression by preventing TAM generation and functions [30]. All these facts suggest that in G4 the remaining TAMs may be predominantly M1, in correspondence with a tumor microenvironment with the high number of IFN-γ^+^ cells seen in this group. To establish if this is the precise mechanism occurring in the tumor microenvironment, a specific identification of each TAMs cell subtype should be carried out.

The third feature observed was the 3-fold higher percentage of PD-1^+^ cells in G4 in comparison with G2. The meaning of an increment in tumors infiltrated by PD-1^+^ cells is unclear and the cause of many scientific discussions, since both antitumor and protumor immune responses have been associated with this increase. For example, studies in melanoma and renal cell carcinoma have related this PD-1^+^ cell increment to T cell dysfunction and poor outcome [31, 32]. But, patients who favorably responded to anti-PD-1 therapy have shown an increased T cells tumor infiltration and PD-1 expression as a first evidence of T cell activation [33, 34]. In concordance with this, we found an antitumor response with high levels of CD8^+^ T and PD-1^+^ cells in G4 relative to G3, possibly suggesting that the anti-PD-1 may reinvigorate tumor-specific T cells already stimulated by the ConvitVax. However, additional functional studies and more specific determinations should be made to accurately address if when applying this combined therapy, the high number of PD-1^+^ cells is indeed a sign of immunosuppression modulated by its interaction with tumor cells expressing PDL-1 or a contribution to tumor elimination through active cytotoxic lymphocytes.

In a general view, the histological results for G4 differed importantly from those reported in G2, which are relevant to have a better perspective of the effects of the different therapies. We found that G4 showed 10% less parenchyma and 10% more necrosis than G2. The mitotic index in G4 was 2.5 times higher than the index reported for G2. Additionally, G4 showed a similar decrease of the MDSCs count when compared to G2. It is possible that the extensive tumor necrosis observed in G4 may have triggered the infiltration of these cells [35], as well as a compensatory mitosis. Finally, G4 showed a tumor growth rate considerably higher, around 10-fold, than that observed in G2. One can attribute this apparent non-regression of the tumors as a result of the high amount of necrotic tissue and immune infiltrating cells, which is a positive result that indicates the killing of tumor cells and consequent death of part of the tumor tissue. However, at the moment of evaluation, the size of the tumor had not yet been significantly reduced. On the other hand, this result could be associated with the high mitotic index, low CD8^+^ T cells percentage and possible presence of Tregs observed in G4, which positions this combination in disadvantage when compared to the stronger immune response observed in G2 [11].

Other relevant processes that may reflect effectiveness or the mechanism of action of the treatment are worthwhile mentioning. For example, the initial step of an effective immune response, which involves the antigen cross-presentation by DCs, generally requires high and stable levels of antigen expression mediated by tumor cell apoptosis/necrosis releasing those antigens [36]. ConvitVax and the combined therapy clearly met this condition, since tumor antigens are a component of the vaccine, and an extensive tumor necrosis was observed. As a consequence of necrosis, different damage-associated molecular patterns (DAMPs) are released and recognized by DCs through pattern-recognition receptors leading to DCs maturation, which is essential for correct T cell priming [37]. The DCs maturation status, estimated by the calculated APC/T cell ratio plus the correlations of CD209b^+^ with antitumor lymphocytes, are indicative of a prevalence of mature DCs in G2, G3 and G4. These results are consistent with previous studies using anti-PD-1 during DCs maturation, in which an enhancement of DCs’ survival and an increase of DCs’ immunostimulatory properties were described [38].

B cells are also important immune cells in the development of a strong antitumor response. A possible predominance of effector B cells relative to Bregs in G2, G3 and G4, as suggested by high CTL/B ratios, could be allowing the establishment of an antitumor microenvironment where B cells can differentiate into short-lived plasma cells (for antibody production), long-lived plasma cells, memory B cells or long-lived memory plasma B cells [39]. The antibodies against tumor antigens secreted by these cells can trigger tumor cells clearance by phagocytosis and/or adaptive immunity, complement-dependent cytotoxicity, chemo attraction of other leukocytes or antibody-dependent cell mediated cytotoxicity [40, 41]. These different mechanisms of tumor elimination, mediated by B cells, may be prevailing in G4 where extensive necrosis and the autologous tumor composition of the ConvitVax assures presence of high tumor antigen levels. This is in part supported by the strong correlation between B cells and necrosis percentage observed in G4, probably as a result of tumor antigen-specific antibodies production.

Other relevant antitumor cells are the NK, which are cytotoxic innate immune cells involved in the elimination of cancer cells [42]. In this study, a significant increment of the percentage of NK cells was only observed in G2. Nevertheless, since it has been reported that anti-PD-1 promotes NK cell activation leading to tumor suppression [43], the activation status of this cell subset should be more carefully evaluated to better identify the effect of the treatments.

We expected that the combination treatment (G4) would deliver a robust improvement in the antitumor effect relative to each treatment alone (G2 or G3). However, the combination as applied in this study, showed a limited number of additional positive effects when compared to ConvitVax alone (G2). Considering that most research utilizing anti-PD-1 as an anti-cancer immunotherapy use doses of 100 to 250 μg of the monoclonal antibody [44–46], we believe that the lack of a stronger synergistic effect for the ConvitVax/anti-PD-1 combined therapy was due to the low dose of anti-PD-1 (50 μg) applied in this work. Additionally, recent investigations with combined immunotherapies emphasize the importance of administering each treatment component at different times to obtain an optimal antitumor response [47]. Furthermore, as the major role of PD-1 is not in the initial T cell activation phase, but rather in the regulation of the immune response of antigen-experienced effector T cells within the peripheral tissues, the combination treatment could have benefited more from the ConvitVax if it had been administered first so as to generate tumor-specific T cells [11, 48]. Once this was achieved, the administration of anti-PD-1 could have protected those T cells from the immunosupressive tumor micro-environment (TME), thus counteracting the immune regulatory mechanisms commonly used in malignancies to escape an effective immune response [49]. For these reasons, in subsequent studies a higher concentration of anti-PD-1 needs to be used, as well as a different timing for each treatment application. Further possibilities would be to combine the ConvitVax with other checkpoint inhibitors or target-specific therapeutic compounds that can further potentiate the effect of our vaccine.

Lastly, other considerations for future research would be to use one or more relevant models where smaller amounts of tumor cells are inoculated and grown over longer periods of time. This approach would also allow other study designs such as to apply the ConvitVax several times to test specific responses to this therapy, then administer the anti-PD-1 in multiple doses and evaluate the response to this staggered treatment.

## MATERIALS AND METHODS

### Mice, tumor cell line, and tumor model used

Six to eight-week-old female BALB/c mice were used. Animals were provided by the Escuela de Medicina José María Vargas (Universidad Central de Venezuela, Caracas, Venezuela) and maintained in their animal facility. The 4T1 cell line was provided by the Cellular and Molecular Pathology Laboratory at IVIC and maintained in the recommended medium. This study was approved by the Bioethics Committee of Escuela de Medicina José María Vargas.

#### Tumor model

1 × 10^6^ 4T1 cells were injected subcutaneously (s.c.) into the mammary fat pad of female BALB/c mice. Tumor induction and preparation of the ConvitVax were performed as previously described by Godoy-Calderón et al. (2018) [11].

### Study treatment groups, measurement of tumor volume and calculation of tumor growth rate

Study treatment groups and procedures: 20 BALB/c female mice were randomly assigned to four (4) groups of five (5) animals each. The four (4) groups were as follows: Group 1 (G1) control treated with PBS; Group 2 (G2) treated with ConvitVax alone (200 μg cell homogenate, 0.0625 mg BCG and 0.02% formaldehyde); Group 3 (G3) treated with anti-PD-1 alone (50 μg/mouse, Ultra-LEAF™ Purified anti-mouse CD279 (PD-1) - Biolegend Cat No. 114110); and Group 4 (G4) treated simultaneously with ConvitVax plus anti-PD-1 (vaccine: 200 μg cell homogenate, 0.0625 mg BCG, and 0.02% formaldehyde + anti-PD-1 50 μg) (Table 1). All treatments were initiated five (5) days post tumor induction and consisted of 100 μL of the corresponding treatment injected once a week for four (4) weeks. The PBS and ConvitVax were injected intradermal on the base of the neck and the anti-PD-1 was injected intraperitoneal. The data presented in this manuscript for groups G1 and G2 was previously published by Godoy-Calderón et al. (2018) [11]. Since all animal groups (G1 to G4) were simultaneously processed using the same 4T1 cell passage and treatment schedule, the data was combined for this publication to compare the treatments and provide a clearer view of the overall results.

**Table 1:** Treatment groups

Group	Treatment
**G1**	PBS
**G2**	Autologous tumor cells homogenate protein (200 μg/mouse) plus BCG (0.0625 mg/mouse), plus formaldehyde (0.02%/mouse) vaccine
**G3**	Anti-PD-1 (50 μg/mouse, Ultra-LEAF™ purified anti-mouse CD279)
**G4**	Autologous tumor cells homogenate protein (200 μg/mouse) plus BCG (0.0625 mg/mouse), plus formaldehyde (0.02%/mouse) vaccine, plus anti-PD-1 (50 μg/mouse, Ultra-LEAF™ purified anti-mouse CD279)

Measurement of tumor volume: After verification of tumor appearance, the tumor volume was measured every four (4) days till 28 days post tumor induction and calculated as described by Feldman et al., (2009) [50] and expressed in mm^3^.

Calculation of the tumor growth rate: The tumor growth rate is defined as the percentage of volume increase relative to the tumor volume four (4) days post induction.

### Histology and immunohistochemistry

Five (5) weeks post-tumor induction, all mice were euthanized, tumors extracted and examined pathologically. Tissues were processed as described by Godoy-Calderón et al. (2018) [11] and stained with Hematoxylin and Eosine (H&E). The immunohistochemistry was carried out according to Mihara et al. (2011) [51] and as described by Godoy-Calderón et al. (2018) [11]. The antibodies used are described in Supplementary Data 2, see Supplementary File. Observation and imaging of immunolocalization was performed according to a modification of Iwamoto & Allen (2004) [52] procedures, as described by Godoy-Calderón et al. (2018) [11] using a fluorescent and light microscope Eclipse E600 (Nikon) equipped with epifluorescence illumination, and a SPOT Flex FX1520 camera (SPOT Imaging). The ImageJ software (version 1.46r) (National Institute of Health, Bethesda, MA, USA) was used for image analysis. Immunofluorescence positive cell counting was carried out in six (6) aleatory areas in one (1) section per specimen per mouse. The total count of positive cells for each antigen was calculated taking into consideration the average cellularity obtained for each group.

### Cellularity

Cellularity was determined by image analysis in five (5) aleatory areas (40×) on H&E-stained sections, in three (3) different sections per mouse (n = 15). The image processing methodology was performed as described in detail by Godoy-Calderón et al. (2018) [11]. Results were expressed as number of cells/1000 μm^2^ [53].

### Mitotic index

The H&E-stained sections were analyzed following the same methodology [54] used by Godoy-Calderón et al. (2018) [11]. Briefly, the sections were examined at total magnification 1000×, using immersion oil to enable the recognition of mitotic figures with high accuracy. Only metaphases, anaphases, and telophases were counted, as well as the number of nuclei. The mitotic index is then defined as the number of mitoses per one hundred cells, expressed as percentage and calculated as follows:

Mitotic index = (Number of mitoses per unit area/ Number of nuclei per unit area) × 100

The counted areas were selected randomly, ensuring that only stroma was present in the observed field.

### Quantification of necrosis and parenchyma in tumors

The necrotic areas and tumor parenchyma were determined in tumor sections stained with H&E as described by Godoy-Calderón et al. (2018) [11], following the protocol proposed by Moffitt (1994) [55].

### Statistical analysis

Kruskal–Wallis non-parametric tests were performed followed by Tukey’s post-hoc test. Pearson’s correlation tests were used to ascertain the associations. The PAST statistical program was used, and statistical significance was met by an α level of 0.05, two-tailed.

## SUPPLEMENTARY MATERIALS FIGURE AND DATA



## Abbreviations

APC/T: Antigen presenting cells/T cell ratio
APCs: Antigen-presenting cells
BCG: Bacillus Calmette-Guérin
Bregs: Regulatory B cells
CTL/B: Cytotoxic T lymphocyte/B cell ratio
DABCO: 1,4-diazabicyclo[2.2.2]octane
DAMPs: Damage-associated molecular patterns
DAPI: 4′,6-diamidino-2-phenylindole
DCs: Dendritic cells
EDTA: Ethylenediamine tetraacetic acid
FDA: Food and Drug Administration
FITC: Fluorescein isothiocyanate
H&E: Hematoxylin and Eosine
IFN-γ: Interferon gamma
IVIC: Instituto Venezolano de Investigaciones Científicas
MDSCs: Myeloid derived suppressor cells
NK cells: Natural killer cells
PBS: Phosphate buffered saline
PD-1: Programmed cell death protein 1
PD-L1: Programmed death-ligand 1
PFS: Progression-free survival
s.c.: Subcutaneously
TAMs: Tumor associated macrophages
TCR: T-cell receptor
TME: Tumor micro-environment
Tregs: regulatory T cells
TRITC: Tetramethyl-rhodamine isothiocyanate.
