# Supplementary material for: Evaluation of a ConvitVax/anti-PD-1 combined immunotherapy for breast cancer treatment
Source: Oncotarget. 2019 Nov 12;10(61):6546–60. doi: 10.18632/oncotarget.27283 (PMC6859918; doi:10.18632/oncotarget.27283)
Supplement: Supplementary file 1 [file oncotarget-10-6546-s001.pdf]

Evaluation of a ConvitVax/anti-PD-1 combined immunotherapy for breast cancer treatment

SUPPLEMENTARY MATERIALS

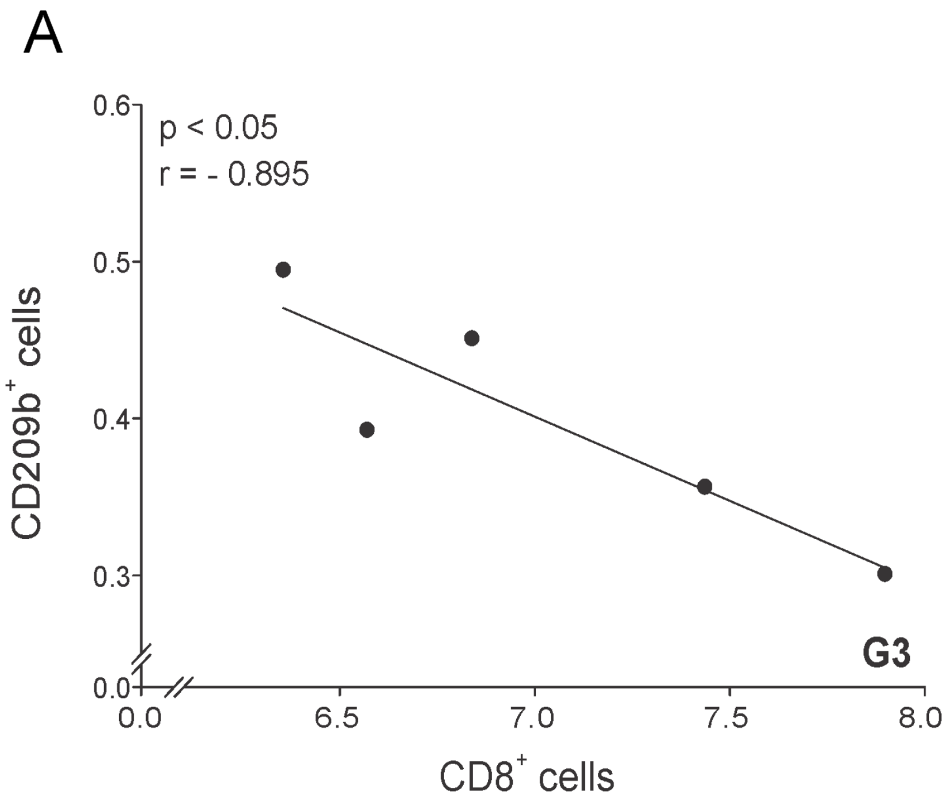

Supplementary Figure 1: (A) Correlation between CD209b$^+$ and CD8$^+$ T cells in G3. The symbol • represents the media ± SEM value for the 5 mice in group G3. The calculated value of the correlation is represented in the scatter diagram (r). For this correlation the *p* value is lower than 0.05.

Supplementary Data 2: List of antibodies used

The following primary antibodies were used: rat monoclonal anti-mouse CD279 (PD-1) (Biolegend, Cat No. 114110), goat polyclonal anti-mouse CD209b (Santa Cruz Biotechnology, Cat No. sc-25221), rat monoclonal anti-mouse CD49b (Biolegend, Cat No. 108901), rat monoclonal anti-mouse CD68 (Biolegend, Cat No. 137001), rat monoclonal anti-mouse CD4 (Biolegend, Cat No. 100505), rat monoclonal anti-mouse CD19 (Biolegend, Cat No. 115501), rat monoclonal anti-mouse CD8-α (Santa Cruz Biotechnology, Cat No. sc-18913), rat monoclonal anti-mouse Ly-6G/Ly-6C (Gr-1) (Biolegend, Cat No. 108401), rabbit polyclonal anti- Integrin αM (CD11b) (Santa Cruz Biotechnology, Cat No. sc-28664) and rat anti-mouse IFN-γ (Biolegend, Cat No.505701). The secondary antibodies used were the following: goat polyclonal anti-rabbit IgG coupled with fluorescein isothiocyanate (FITC) (Abcam Inc., Cat No. ab6717), goat polyclonal anti-mouse IgG labeled with tetramethyl-rhodamine isothiocyanate (TRITC) (Abcam Inc., Cat No. ab6897), sheep anti-rat IgG labeled with TRITC (Abcam Inc., Cat No. ab6849), goat polyclonal anti-rabbit IgG coupled with FITC (Abcam Inc., Cat No. ab6717), mouse polyclonal anti-goat IgG coupled with FITC (Santa Cruz Biotechnnology, Cat No. sc-2356), and goat polyclonal anti-rabbit IgG coupled with Texas red (Santa Cruz Biotechnology, Cat No. sc-2780).
